# Imperatives of recognising the complexities: gendered impacts and responses to COVID-19 in India

**DOI:** 10.1007/s40888-021-00242-8

**Published:** 2021-09-25

**Authors:** Bina Agarwal

**Affiliations:** Global Development Institute, University of Manchester, Arthur Lewis Building, Oxford Road, Manchester, M13 9PL UK

**Keywords:** Gender impact, COVID-19, Direct & indirect effects, Surveys & data gaps, Rural–urban, Building back better, India, J16, O15, O53

## Abstract

This paper argues that the gendered impact of COVID-19 has both visible and hidden dimensions, and both immediate effects linked with lockdowns and longer-term effects that are likely to emerge sequentially in time and affect recovery. Much of the existing feminist literature on the impact of COVID-19 has neglected these complexities and focused mainly on care work and domestic violence. This has diverted attention away from other key concerns such as livelihood loss, food and nutritional insecurity, indebtedness, rising poverty, and the low resilience of most women in developing economies. Even care work and domestic violence have complex facets that tend to be missed. Using examples from India, the paper outlines the kinds of gendered effects we might expect, the extent to which these have been traced in existing surveys, and the data gaps. It also highlights the potential of group approaches in enhancing women’s economic recovery and providing social protection from the worst outcomes of the pandemic—approaches that could guide us towards effective policy pathways for ‘building back better.’

## Introduction

To understand the gendered impact of COVID-19, we need to focus both on the visible dimensions of the impact and the hidden dimensions; and both on the immediate effects of the pandemic and lockdowns, and on the medium and long-term effects which are now starting to emerge and could worsen with time. These delayed effects can arise due to women’s inability to recover from the troughs they encountered in the immediate onslaught of the pandemic in terms of livelihood loss, which then spill over to food insecurity, depletion of savings, reduction in credit-worthiness, a decline in intra-household bargaining power (Agarwal, 1997), and so on. Much of the global focus, however, has largely been on two gender effects—increase in care work and domestic violence.^1^ These two effects, while important, fail to cover the full impact on women, or to factor in impact *as a sequential process* rather than as a one-time event. Also a persistent focus on just two dimensions diverts policy attention away from other critical aspects of women’s well-being in developing economies.

Using India as an illustration, this paper first outlines what kinds of gendered outcomes we can expect, drawing on our knowledge of pre-existing gender inequalities and people’s coping strategies under other types of crises (see also, Agarwal, 2021a, 2021b). It then examines COVID-related evidence to see to what extent these potential effects are captured by existing surveys, and the data gaps that remain. It also highlights group approaches through which women have remained, or can remain, better protected from the worst economic outcomes of the pandemic, and point to ways forward for shaping effective future policies.

## Anticipating complexities

### Expected immediate and direct effects

The most direct and immediate effect expected from the pandemic and associated lockdowns was on jobs and livelihoods. But beyond aggregates, we would expect the impact to differ according to the nature of employment (formal or informal), the sector of work (agriculture, industry or services), its location (rural or urban), and the type of job (salaried, self-employed or casual). Although both men and women in India are working largely in the informal sector which employs 90% of female workers and 86% of male workers (GoI, 2019), the informal sector itself is very diverse in terms of earnings. There are also substantial gender differences in other respects. To begin with, women are concentrated in rather few occupations. In rural India, 75% of women workers are in agriculture relative to 55% of male workers (GoI, 2019); and, within agriculture, most women provide unpaid labour on family farms or are low waged agricultural labourers and artisans. In urban areas, many are located in the lower-earning, insecure end of the spectrum, such as street vendors or domestic workers, and only some are in the more secure end working as teachers (mainly in primary schools) or nurses, with the rest located in-between, such as in manufacturing and construction.

Overall, then, compared with men, women have fewer livelihood options, are in lower paid and less secure jobs or in unpaid work, and some of the growing urban jobs, such as of domestic helps, are ‘touch heavy’ and thus at particular risk in a pandemic. Women are also less mobile, both across occupations and physically (due to domestic responsibilities and physical insecurities). Once displaced, they are thus less likely to recover economically. If they were migrants from rural areas, those who have gone back are less in a position to return and find employment and accommodation easily, as in the case of domestic workers. The self-employed, similarly, may be unable to invest in stocks or productive inputs.

### Expected sequential effects

Joblessness, in turn, can have a sequence of effects. Especially in urban contexts, lockdowns can lead to food insecurity and hunger, given a lack of funds to procure food (Scroll, 2020). To subsist, we would expect the jobless to draw first on savings, then borrow from friends, relatives, banks, and moneylenders, and finally begin to sell assets (Agarwal, 1990). From existing research on the coping strategies of rural communities during climatic disasters such as severe droughts, we note that households facing food shortages tend to first reduce consumption and deplete stocks of grain, fodder, and so on, before selling productive assets on which their long-term prospects of recovery depend (Jodha, 1975). There also tends to be a sequencing in asset disposal: households usually first dispose of smaller assets, such as minor animals or pieces of jewellery, while holding onto substantial assets such as cattle and land as long as possible (Agarwal, 1990; Jodha, 1975).

These sequential effects are likely to vary by the economic class of the household. Households that are well endowed with savings and productive assets can tide over the crisis. Households with few savings and assets can lose them over time, depending on how long it takes to restore jobs and livelihoods; while households with no savings or saleable assets would fall into poverty, unable even to borrow due to reduced credit-worthiness.

Women, however, are likely to be affected differently from men. To begin with, in developing economies, they are working in a narrower range of jobs and have fewer savings and assets to draw upon, even in the better-off households. Barely 14% of rural women own land in India, even in rural landowning households (Agarwal et al., 2021). Moreover, the smaller assets, such as small animals and jewellery, which tend to be sold first under crises, are usually assets owned by women, while the assets that are retained, such as land and large animals, are usually owned by men (Agarwal, 1990). Selling small items may be logical in economic terms, since land and cattle are the means of production for farming communities. But this sequence of sales can have hidden gender costs, since women’s bargaining power within the home is undermined if they lose their assets while men retain theirs, leading to adverse consequences, including the abandonment of women or girls, as discussed later (Agarwal, 1990).

Ironically, those with secure jobs, such as women healthcare workers, can be vulnerable in other ways in a pandemic. For example, they could be more at risk of infection than men since they are less likely to get scarce protective gear under a professional hierarchy which prioritises doctors, 84% of whom are men in India, over nurses, 83% of whom are women (Anand & Fan, 2016).

### Expected indirect effects

A range of other indirect and hidden consequences can follow for women due to gendered social norms and shifts in intra-household power relations. This can affect both paid women workers and those unpaid but working in family enterprises.

To begin with, women are likely to be affected by a reduction in household incomes not only due to their own loss of jobs but also if their spouses or other male household members lose jobs locally, nationally or internationally. The return of male migrants to villages can also lead to an overcrowding of limited local jobs for women.

Second, food scarcity is likely to affect women and girls more since in many regions, under existing social norms, they are expected to eat last and least. We can thus anticipate gender inequalities in diets and nutritional inequality within homes. This may at best be mitigated but not eliminated by government relief measures, as discussed in the concluding section.

Third, while women may have some inherent biological advantage that reduces fatalities in pandemics (in most countries more men than women have died from COVID-19: Global Health 50/50), this advantage could get negated by health conditions that put particular categories of women at high risk.^2^ There is widespread global recognition, for instance, that pregnant women face higher risk in pandemics. This is especially likely in India, not only from the virus but from poorer nutrition and the restricted hospital care available for medical needs unconnected with COVID-19.

In addition, Indian women have a higher incidence of comorbidities—malnourishment, anaemia, and obesity—than men, especially due to longstanding intra-household nutritional inequalities. In 2015–2016, studies based on nationally representative data found that 22.4% women relative to 19.6% men were underweight (Dutta et al., 2019), and 53.2% women in the 15–49 age group while 23.2% men in the 15–54 age group were anaemic (Didzun et al., 2019). The relative incidence of overweight/obesity was 20.7% for women and 19.6% for men (Shannawaz & Arokiasamy, 2018). Moreover, as of 2014, while women and men aged 30 or over had the same prevalence of heart disease, ‘women were likely to seek care at lower levels of service provision, even though they carried a higher level of multiple morbidities’ (Sandeep et al., 2018: 1). Pre-existing gender biases in health care access has been noted too in other pandemics (Kumar & Quin, 2012; Sandeep et al., 2018).

Hence, although Indian women may be less prone to dying under COVID-19 *on average*, certain categories of women are more vulnerable than men. In fact, in the 1918 Influenza pandemic, excess female mortality^3^ among Indian women was higher than among males, *both* on average (the reverse of other countries) and among females of 5–40 years, and especially among those in the reproductive age group of 20–40 years (GoI, 1920; Murray et al., 2006). Insightfully, the 1918 *Report of the Sanitary Commissioner* (GoI, 1920, 63) attributed this to two factors: the disease being exceptionally fatal for pregnant women, and women being caregivers in sick households while themselves being sick (in COVID-19 parlance they had high viral load from repeated exposure).

Fourth, the higher death rate among men than women under COVID-19 itself has indirect consequences for women which I have not seen discussed anywhere, namely the economic and social precarities women can face as widows, as elaborated further below.

Fifth, consider the much-discussed impact on care work. A 2019 all-India Time Use Survey (GoI, 2020a) reports high pre-COVID gender inequalities, with females and males respectively spending on average 5 h and 1.6 h per day in unpaid domestic work (rural–urban differences are small). Under COVID-lockdown, as out-of-school children and jobless husbands stay home, women’s domestic load can be expected to increase. In urban households that had earlier employed part-time domestic help, the absence of this external support can increase care work time. Professional couples may share the extended domestic tasks to some extent, but in most households care work falls largely on women’s shoulders. In rural India, this can also mean more time and energy spent on fetching water, gathering firewood (which remains the single most important source of fuel in South Asia and sub-Saharan Africa: Agarwal, 2010), collecting fodder (animal care is largely women’s work), and foraging for supplementary food items from local forests and commons (Agarwal, 2010). Much of the global discussion on women’s care work under the pandemic relates to urban middle-class nuclear homes in the Global North rather than extended rural households in the Global South.

Sixth, male unemployment and the concentration of family members in small spaces can increase women’s risk of domestic violence (both verbal and physical). More generally too, male unemployment is found to be associated with a higher incidence of domestic violence (Agarwal & Panda, 2007). In other words, women can be affected not only by their own loss of employment and earnings but also by the unemployment of their spouses. Again we would expect diversity among women, in terms of class. Those owning immovable property, such as land or a house, are found to be at dramatically lower risk of domestic violence than propertyless women (Agarwal & Panda, 2007). Owning a house or land can deter violence because they give a woman a credible exit option, or, if the couple is living in her house, she can ask her husband to leave.

Economically better-off women would also be better placed to seek help during the pandemic when faced with violence, since they can connect with helplines through their cell phones, while poor women lacking individual cell phones would remain at high risk. In India, a 2019 study of 2000 rural and urban adults found that 63% of the women relative to 79% of the men owned mobile phones; and only 21% of the women relative to 42% of the men had mobile internet (GSMA, 2020). In other words, we need to move beyond cross-class generalisations about the impact of the pandemic on domestic violence to a more intersectional approach by women’s class and property status.

Seventh, in poor households, another form of violence against women may emerge which is little talked about. For example, in the Great Bengal Famine of 1943–1944, when poor women’s limited employment options collapsed, many women were abandoned or pushed out of the home by spouses, and daughters were sold off into prostitution, even where men retained their small plots of land or had other earning options (Greenough, 1982). This may seem too dramatic to imagine in the twenty-first century in the context of the current pandemic, yet it cannot be ruled out as a potential outcome, as discussed further below.

Eighth, we can expect both gender and class inequality in access to online learning with school closure. Large numbers of households do not have computers or mobile phones with internet access. In households with only one phone, we would expect boys to get priority over girls for online classes and other school work. Girls may even drop out of school altogether and not return (Nikore, 2020).^4^

**Fig. 1:**
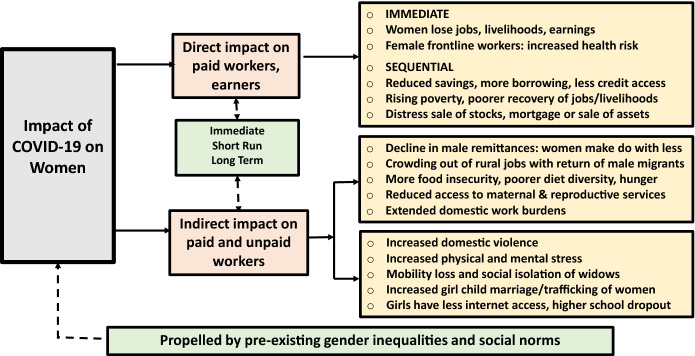
Gender impact of COVID-19: Direct and indirect, immediate and long-term. *Source*: Based on current paper. Figure format adapted from an earlier figure constructed by A. K. Shiva Kumar and Bina Agarwal which was based on Agarwal (2021b)

Many of the above gender effects are likely to play out not only in the immediate short-term but also in the medium and long-term, since women tend to have much less resilience than men, both economically and socially. Figure 1 attempts a schematic representation of these potential effects. It is only a broad representation of some of many possible visible and invisible gendered effects we might expect. More could be added (or some subtracted) by context and region, given variations in social norms and pre-existing gender inequalities across India, especially between the northern and southern states. Also, what may be an immediate effect for a poor urban daily earner (such as food shortages) may not be felt immediately in a household which has some savings, or in a farm household that is cultivating food crops. As discussed in the section below, however, even the types of impact outlined above have been missed in large part in existing, typically one-time surveys, with some limited catching up by a few recent studies.

## What did surveys capture and what is missing?

From late March 2020, many organisations in India carried out telephone surveys, a few with large samples and covering many states but most with small samples and often focused on one region or one occupation. I tracked 23 surveys and found that barely 11 had covered any aspect of gender, and those that had done so had typically covered only one dimension (see Agarwal, 2021b). Some surveys were focused only on women, with no comparative data on men. And most were limited to one point in time, giving a snapshot of a given moment in the pandemic but not developments over time. Indeed, it is news reports and the media, the so-called ‘grey literature’ which academics typically eschew, that have provided more nuanced leads on women’s experience of the pandemic. But hardly any of these leads were followed through by large-scale longer-reach surveys.

Notably too, since women respondents often lack personal mobile phones or privacy, gender-related telephone surveys need to factor in the likelihood of family members listening in (Alvi et al., 2020). This can lead to underreporting not only of domestic violence but also of intra-household inequalities in the distribution of workloads and food. With these caveats, consider what we can learn about the impact of COVID-19 on Indian women from existing evidence, on the various counts outlined.

Broadly, as seen from Table 1,^5^ the issues that have been best covered by systematic surveys (as vs. media reports) undertaken in 2020 are the immediate direct effects on women’s employment and women-directed government relief schemes, while the most neglected are the indirect intra-household effects. A few gaps are only now being filled by recent studies. This has been made possible (a) by the availability of periodically collected panel data which predate the pandemic and have continued during the pandemic—these enable comparisons between 2020 and 2019—and (b) through surveys tuned to catch the impact on particular sections, such as small farmers and single women, or particular aspects, such as food security and diets (see e.g. Gupta et al., 2021; Harris et al., 2020; Kulkarni et al., 2021). These latter surveys on rural India demonstrate how normally hidden dimensions can be captured if there is prior recognition of gendered vulnerabilities.

**Table 1:** Gender Impact of Covid-19 in India: sources of information, if any

Nature of impact	Source of information	Sample size	Region
**Employment**			
Women and men	Deshpande (2020, 2021), using data from Centre for Monitoring the Indian Economy (CMIE)^b^	c.50,000 persons, April, August, December 2020	All-India, panel survey
Women and men	Abraham et al. (2021), using CMIE Consumer Pyramids Household Survey (CPHS)^c^	22,330 persons (trajectory sample) Dec 2019, April, August 2020	All-India, panel survey
Women and men	CSE: Azim Premji University^d^	4700 households, April–May 2020	10 states, and some cities, periodic
Women, SEWA members, mostly street vendors	SEWA-Bharat (2020)^e^	300 women in April–May 2020	11 states
SEWA members	IFPRI-SEWA^f^	600 women, May–June 2020	Gujarat state
Women, informal sector workers	ISST, Delhi^g^	176 women	Delhi state
Women farmers (widowed/single)	Kulkarni et al., (2021) primary survey^h^	946 widowed/single women farmers, 17–25 May 2020	Maharashtra state
Women, group farms	Kudumbashree (2020a)^i^	c. 30,000 women’s group farms, April 2020	Kerala state
Women, micro-enterprizes	Kudumbashree (2020b)^j^	1015 enterprises, 2020	Kerala state
**Savings/assets**			
Women’s loss of savings, borrowing	SEWA-Bharat^e^	300	12 states
Women’s loss of assets	None	–	–
Impact of remittance loss	None	–	–
**Health**			
Pregnant and lactating women	Counterview desk^k^	R1: 87 women, 26–31 March 2020 R2: 40 women, 4–11 April 2020	6 states
Healthcare workers, Covid infections, deaths^a^	None	–	–
Covid infections & deaths by age & gender^a^	None	–	–
**Food & nutritional diets**			
Gender specific food security, diets, hunger	Gupta et al. (2021)^l^ (a) CMIE/CPHS (b) Plus primary survey	CMIE/CPHS, May 2019, May 2020 Primary: 3600 HHs, May 2019, May 2020	All-India 4 districts in 3 states Bihar, UP, Odisha
Gender specific diet diversity	Harris et al. (2020), primary survey of farmers^m^	448 farmers, May 5–12, 2020	4 states: Assam, Jharkhand, Andhra Pradesh, Karnataka
**Care work, violence, widowhood, girlhood**			
Care work	CSO (2020)^n^	R1: 5162 rural HHs, April–May 2020 R2: 4835 rural HHs, June 2020	12 states (Round 1); 11 states (Round 2)
Domestic violence	Media reports^o^ and National Commission for Women^p^	–	10 states
Widowhood	Author initiated survey with colleague	13 widows, September 2020	Rohtak district, Haryana state
Early marriage, sale of girls	Media reports^q^	–	–
Education of girls & boys	None	–	–
**Government relief measures**			
Cash transfers to women (PMJDY)	Dvara Research^r^	322 microfinance HHs, April 2020 (Round 1)	9 states
Various relief measures	CSE (2020)^d^	2670 persons, April–May 2020	10 states, 2 cities
Various relief measures	Kulkarni et al. (2021)^h^	946 Widowed/single women farmers, 17–25 May 2020	Maharashtra state

Source: Compiled by the author from various sources [all links were accessed on 13 August 2021]

HH:  household, *R1*,* R2*: rounds 1 and 2

^a^Global Health 50/50 (2020) has this information for some countries, but not for India

^b^Deshpande (2021). https://doi.org/10.1007/s40888-021-00235-7; https://consumerpyramidsdx.cmie.com/

^c^Abraham et al. (2021). https://doi.org/10.1007/s40888-021-00234-8

^d^CSE (2020). https://cse.azimpremjiuniversity.edu.in/covid19-analysis-of-impact-and-relief-measures/

^e^SEWA-BHARAT (2020). http://www.sewabharatresearch.org/wp-content/uploads/2020/05/Gendered_Precarity_SB_Lockdown-1.pdf

^f^IFPRI-SEWA (2020). https://pim.cgiar.org/2020/07/14/phone-surveys-to-understand-gendered-impacts-of-covid-19-a-cautionary-note/

^g^ISST (2020). https://www.isstindia.org/publications/1591186006_pub_compressed_ISST_-_Final_Impact_of_Covid_19_Lockdown_on_Women_Informal_Workers_Delhi.pdf

^h^Kulkarni et al. (2021)

^i^Kudumbashree (2020a). https://www.kudumbashree.org/storage//files/qdzl7_agri%20covid19.pdf

^j^Kudumbashree (2020b). http://www.kudumbashree.org/storage//files/vhs7r_me%20study%20report-sajith.pdf

^k^https://www.counterview.net/2020/04/were-daily-wagers-how-will-it-be-okay.html

^l^Gupta et al. (2021). https://link.springer.com/article/10.1007/s40888-021-00233-9

^m^Harris et al. (2020). https://link.springer.com/article/10.1007/s12571-020-01064-5

^n^CSO. http://www.vikasanvesh.in/wp-content/uploads/2020/06/Presentation-based-on-CSO-consortium-survey.pdf

^o^Indian Express (2020b)

^p^http://ncw.nic.in/ncw-cells/complaint-investigation-cell

^q^CNN News^18^. (2020). https://www.news18.com/news/buzz/child-marriage-or-trafficking-choice-covid-19-and-cyclone-amphan-have-left-for-bengals-vulnerable-2780323.html

^r^https://www.dvara.com/research/social-protection-initiative/covid-19-impact-on-daily-life/

### Direct effects: immediate and sequential

Let us begin with employment. Only one systematic Indian survey—conducted by the Centre for Monitoring the Indian Economy (CMIE)—has provided periodic panel data under the pandemic for all-India, covering over 50,000 households. Analysed by Deshpande (2020, 2021) for three rounds in 2020 (April, August and December), we know that although in absolute terms, in April 2020, men lost more jobs than women (given that more men than women were employed), women were 20% points less likely to be employed among those employed prior to the pandemic. And in August 2020, while both women and men recovered compared to April, the recovery was partial for both but much lower for women than men.^6^ Subsequently, employment declined for both sexes, even as gender gaps in employment returned to the December 2019 levels. The CMIE data do not cover the number of workdays and earnings. Hence we cannot assess if even those employed are earning enough for subsistence or have simply joined the ranks of the ‘working poor’.

What the CMIE does provide, however, through its Consumer Pyramid Household Survey, are data for more in-depth analysis of the gendered impact of the pandemic at the household level. Abraham et al. (2021) who analyse these data provide additional insights, such as the divergent gender effects linked to education and marriage. They find that while education protected men from job loss, highly educated women were *more* vulnerable to job loss; and while married men were more likely to return to work, married women were less likely to do so. Moreover, while men who returned to work were able to find alternative earning options through wage work or self-employment, women did not have the same mobility across diverse earning options and were therefore often forced to quit working.

Studies based on small regional surveys argue that wage-earning women were more affected than the self-employed (Desai et al., 2021), and women in cities were more affected than those in villages. This appears to be true, but only to a limited extent. In urban India, for example, large numbers of the 4.75 million domestic workers, of whom women constitute some 3 million (ILO, 2020), lost their jobs, and many have not been hired back for fear of infection from this ‘touch heavy’ occupation (ISST, 2020). In contrast, in the villages, women could still find work in agriculture and under the Government of India’s Mahatma Gandhi National Rural Employment Guarantee Scheme (MGNREGS) which guarantees 100 days of employment per rural household annually. However, this broad urban–rural narrative needs some interrogation.

In MGNREGS, for example, women faced competition from returning male migrants. In addition, recent surveys focused specifically on rural India indicate that even in villages, and even among self-employed women farmers, losses were heavy. For example, Harris et al.’s (2020) study in May 2020 of 448 farmers who were part of the World Vegetable Centre project in four Indian states found that a vast majority of the farmers faced serious decline in vegetable sales, prices and farm incomes. In 90% of the farms income fell, and in 60% it fell by more than half. The losses suffered by women farmers were higher than for men.

Similarly, Kulkarni et al.’s (2021) study of single women farmers (mostly widows) in Maharashtra, in western India, found that women lost their crops due to lack of harvest labour and markets, and those who could sell had to do so at much lower prices than pre-pandemic levels. Many could not access transport to take their produce to the market at all, or found the prices charged to be unaffordable. As a result of the loss, they could not repay their loans taken from informal credit sources at high interest rates. This reduced their credit-worthiness. Further, unable to borrow they could not invest in seeds and other inputs needed for the next production cycle. Some said they might now lease out the land or keep it fallow. In other words, an immediate adverse impact can set in motion a declining cycle of earnings, with each cycle of income reduction lowering the likelihood of recovery. This further highlights the sequential effects I had mentioned earlier.

Moreover, 10% of Indian rural households depend for over 30% of their annual household expenditure on domestic remittances from migrants (Tumbe, 2011), and many more depend on international remittances. These flows have been largely cut off under COVID-19 restrictions (Reja & Das, 2020). Although we know rather little about the impact of this loss on the dependent households, and especially the impact on women, we can expect women to bear the burden of making do with less.

As people lost jobs and livelihoods, families drew first on savings and then borrowed from relatives and moneylenders (CSO, 2020; SEWA-Bharat, 2020), but this information is not gender-disaggregated in the CSO survey which has a large sample, and is available only for women in the smaller SEWA-Bharat survey which covered members of the Self-Employed Women’s Association (SEWA).^7^ The latter study found that 91% of the 300 self-employed women interviewed had used up their savings and borrowed from relatives and friends and 9% had borrowed from moneylenders. We do not know, however, if the SEWA women needed to use their own collateral or their family’s collateral when they borrowed. Did their spouses or other males also have to borrow, or were these figures only for women?

Next, on the impact on assets, there is a consistent absence of detailed information, although two among the ongoing surveys agreed to ask additional questions at my request. Of these, SEWA-Bharat recorded no notable asset sales beyond the occasional mobile phone (where families had more than one), in the immediate months following the pandemic. But female street vendors when asked what they would prioritise if they needed to sell assets said that they would first sell unsold stock and animals before considering carts or rickshaws (SEWA-Bharat, 2020). These surveys were not repeated, but it is clearly essential to follow the trajectory of asset disposal. In this context, a report by Reuters is strongly indicative: it reports that many families, including farmers, used gold as collateral to get loans from banks which were otherwise unwilling to lend in the pandemic (Anand & Jadhav, 2020). It is quite likely that this gold was in the form of women’s jewellery. Also, in a documentary film by Rangan Chakravarty on the impact of COVID on poor rural women in West Bengal in 2020, some women reported selling their jewelry to survive (Chakravarty, 2021). More generally, sales of assets can mark a slide into poverty. Poverty numbers in India are estimated to have increased by 75 million due to the economic downturn linked with COVID-19. There has also been a large shrinkage in the numbers of middle-class households, according to a Pew Research Centre analysis (Kochhar, 2021).

### Indirect effects, often hidden within families

Indirect gendered effects arise as responses to the direct ones, not only due to pre-existing inequalities but also due to changing intra-household gender dynamics. This complexity can make these effects less obvious and even invisible. By way of illustration, included among such effects is a rise in food and nutritional insecurity and hunger; difficulties faced by pregnant women in accessing medical services; increase in care work burdens and domestic violence; abandonment of young women and early marriage for girls; insecurities and dependencies experienced by widows whose husbands have died from COVID; and educational reversals for young girls. Consider some examples.

The most serious immediate impact of the pandemic has been on access to food. While in early 2020 several one-point surveys and media reports focused on growing food insecurity and hunger under the lockdown, none provided gendered insights. Even the earlier-mentioned CSO (2020) survey for rural India did not tell us the relative sacrifice of male and female family members, although it highlighted a reduction in the number of daily meals and items eaten per meal during the pandemic.

In this context, a recent study by Gupta et al. (2021) is a refreshing change. Based on primary and secondary data sets, it examines the pandemic’s impact on both household food availability and women’s dietary diversity at the national, state and district level—the last in four economically backward districts of three states in north and east India. It finds a decline in household food expenses at all regional levels in May 2020 relative to May 2019, despite public distribution of grains and government cash transfers. Notably, the study finds a disproportionate decline in women’s consumption of non-staples like meats, eggs, vegetables and fruits. Other studies reinforce this. Harris et al. (2020) who focused specifically on vegetable farmers found that women farmers were much more likely to report a substantial reduction in the consumption of vegetables, fruits and dairy, especially due to unaffordable prices. And Kulkarni et al. (2021) found that single women farmers in Maharashtra reported food scarcity and were thinking of shifting from cotton to food grains to gain some food security in future crises.^8^

These gender inequalities in nutrition under the pandemic are likely to show up in longer-term gender disparities in health outcomes, especially for reproductive age women and lactating mothers. In fact, some qualitative surveys and media reports on small numbers of pregnant and lactating women found that they faced a range of difficulties, including a lack of rations for adequate nutrition (National Law School of India, 2020). Poor women were also unable to reach hospitals for delivery due to transport restrictions during the lockdown (Bisht et al., 2020), but we do not have a systematic tracking of such cases. And women healthcare workers who, as noted earlier, tend be lower in the medical hierarchy than men, were reported to be less likely to get protective gear (Times of India, 2020).

Another indirect effect on women has been the much-discussed care work burden. Here, information on the impact on *rural* women’s care workloads in India is again sparse. A rare exception is the earlier-mentioned survey by CSO (2020) which covered 4835 rural households across 11 states in June 2020 and found that of the 820 returned migrant households for which there was information, 53% and 71% reported an increase in women’s time spent in fetching water and firewood respectively, the comparable figures being 39% and 52% among non-migrant households. Among urban middle-class couples, men shared some domestic tasks under lockdown, but reverted to pre-COVID patterns when they returned to work, while women’s average hours spent on domestic work increased sharply (Deshpande, 2020, 2021). Also, what is not captured in time use data is the *responsibility* women feel for ensuring food security for the family. In rural India, for instance, women were found to report high levels of anxiety due to their inability to continue feeding their families under the national lockdown (Dhawan et al., 2020).

Similarly, we know that domestic violence increased during the pandemic lockdown, but the figures available are likely to be gross underestimates, since India’s National Commission for Women, which recorded domestic violence under the lockdown, depended on emailed complaints: this excluded poor urban women and most rural women who do not have access to internet and may not even know how to use email, apart from the lack of privacy in small homes which would prevent them from reporting violence. None of the figures are divided by women’s class or caste. The extent of verbal abuse and the implications of both physical and verbal violence on women’s mental health have also been little examined. Other forms of abuse have surfaced, however, but only through the media, such as nurses being harassed by men in isolation wards (Indian Express, 2020a).

Of particular concern are reports of a very different type of gender abuse, namely the early marriage or sale of girls among poor households, reminiscent of the effects observed during the Great Bengal famine, as mentioned earlier. Again, this was not captured by research surveys, but were reported by the media based on child distress calls tracked by the Ministry for Women and Child Development. Between March and August 2020, the Ministry tracked 192,000 interventions compared to 170,000 in the same period last year (Indian Express, 2020b). There were also emerging reports of young women being rescued by the police from dance groups to which they had been sold off (many such groups are linked with prostitution) (CNN news18, 2020). Due to social stigma, families seldom accept back these “rescued” girls who then end up in women’s shelter homes which too can be dens of sexual exploitation.

Notably, neither the surveys nor the media have reported an important indirect effect, namely that on women who have been widowed under COVID-19. I therefore initiated a small qualitative survey with a colleague, Kavita Chakravarty, to interview women whose husbands had died from COVID in the Rohtak district of Haryana state in north-west India. Of the 15 women so widowed in the District (by the Records of the Rohtak District Chief Medical office), 13 (3 rural and 10 urban) could be interviewed telephonically in September 2020. Over half had applied for but had not yet received their widows’ pensions which, in any case, are inadequate (Jan Sahas, 2020). Four of the widows had been married to men with government jobs and were awaiting their husband’s pensions, and two were well-off. Only three, all of them poor, had jobs themselves (a domestic help, an agricultural labourer, and a tailor). For middle-class women, however, the most deeply felt issue was not economic but reduced social independence and mobility. Most reported that they were now dependent entirely on their adult children, especially sons, to accompany them on social visits or to go outside the city.

Box 1 provides excerpts in their own voices. W7, a retired school teacher, for example, said: ‘If I need to go out I have to depend on my son. He has his own family, so he will take me only at his own convenience.’ That even a former school teacher should feel so dependent on a son for social mobility may seem surprising, but underlying this response are social norms and the gender segregation of public space—notionally if not formally—in conservative communities (Agarwal, 1994), which can deter widows from making social visits on their own. Also, married at young ages, middle-class women typically build their lives around the spouse and his family and friends, with few independent friendships to fall back on. The only widow (W4) who felt socially connected was a member of a small women’s temple group. She reported: ‘Earlier we used to go for day pilgrimages together … I can resume that again after Corona.’ But even she added: ‘If I need to go anywhere else, my son will take me.’

While reduced mobility can be seen as a general widowhood penalty in conservative social contexts, COVID-19 has worsened it, given the restrictions on movement in general, including limited options of public transport. At the all-India level too, time spent with friends is found to have fallen much more sharply among women (not specifically widows) than men, and had declined to about one-third of pre-pandemic levels by December 2020 (Deshpande, 2021).

In discussing the effect on women, the impact on girls and young women cannot be forgotten. Here, although there is concern expressed in the media on the negative effect of the pandemic especially on the education of girls (Sonawale, 2020)—by some estimates up to 10 million girls are likely to drop out of secondary school (Trivedi, 2021)—none of the surveys I saw had followed this up or built it into their questionnaires. The long-term impact can be worse for girls than boys, since girls are less likely to recover, especially in poor homes with little or no internet or access to online classes. And this could also affect their future prospects of entering into higher education institutions. There is evidence (although ungendered) found in medical journals, of the adverse impact of school closures during the pandemic on the mental health of children and adolescents (Patra & Patro, 2020).

Overall, therefore, gender-disaggregated information on the impact of the pandemic is at best patchy. Most surveys are one-time only and seldom focus on intra-household effects or identify intersectional differences by class or caste.

The one issue which did get coverage in several surveys in 2020 was the government’s relief packages in cash or kind, especially the Rs. 500 ($ 6–7) per month promised specifically to poor women for three months (later extended) under the Pradhan Mantri Jan-Dhan Yojna (PMJDY). All the surveys that examined this found that the relief did not reach the majority of women. One survey of 2670 respondents conducted during mid-April–May 2020 across 10 states and two cities found that 64% of eligible women did not get the cash relief as they lacked the special PMJDY bank accounts to which the transfers were to be made (CSE, 2020; see also Dvara, 2020; Pande et al., 2020 and Dhawan et al., 2020). Also, many women could not withdraw the cash from banks, located many kilometres away, due to a lack of public transport during lockdown and the high cost of private transport which could come to 20–30% of the cash relief promised (Kulkarni et al., 2021).

Other government schemes too had hidden barriers. For example, there was a scheme for providing poor households with three cooking gas cylinders during the pandemic. Funds to pay for them were to be advanced to women’s bank accounts, but many women did not receive the advance, or were not listed as potential beneficiaries in the first place (Kulkarni et al., 2021). Later the scheme was changed and required women to first purchase the third cylinder and get reimbursed later, based on receipts (Business Standard, 2020). The scheme thus had limited effectiveness, since many households were excluded, and those included could not afford the cost of the third cylinder. Another central government scheme of making cash transfers of Rs. 6000/year to farmers owning under 2 ha of land could not benefit most women farmers, given that few of them own land in India.

Almost all surveys, however, focus on the negative effects of the pandemic, with rather little on the positive side. Yet these are equally important to document.

Box: Interviews with widows whose husbands died from COVID-19, Rohtak district, Haryana, September 2020 Excerpts from answers to the question: *How do you see the future economically, socially and emotionally?*

W1. [Rural, upper caste, 62 year old housewife with 10 years of schooling, was married for 46 years to a man who retired from government service]. ‘I am estranged from my son. I now live alone with my college-going granddaughter. If I have to go out, my daughter comes over. What can I say about my earlier life? It was so different. I could go everywhere with my husband if I needed to… Just the absence of one man can totally overturn your life’ (starts crying).

W2. [Rural, scheduled caste, 44 year old unschooled agricultural labourer, was married for 24 years to an agricultural labourer]. ‘I used to work as an agricultural labourer even earlier and I continue to do so now. I can easily go in and out of the village, but, yes, if I want to visit relatives in another village I have to depend on my sons who are not yet married. In any case, I have to work to feed myself. We have exhausted our savings and I have borrowed from our neighbour.’

W3. [Rural, backward caste, 42 year old housewife without schooling, was married for 24 years to a poor barber]. ‘My husband was always there to support me if I wanted to go out. Now I am dependent on my children. My son who was studying for B.Com in a college in Rohtak is now working as a construction labourer…We used up our savings on my husband’s illness and for subsistence.’

W4. [Urban, upper caste, 58 years old with 5 years of schooling, was married for 39 years to a shop owner]. ‘We have a shop on the ground floor. To pass the time, I go sit in the shop. In the temple we have a small women’s group so I also go there. Earlier we used to go for day pilgrimages together. I can resume that again after Corona. If I need to go anywhere else my son will take me.’

W5. [Urban, scheduled caste, 39 years old with no schooling, worked as a domestic help, was married for 23 years to a sweeper on contract]. ‘My husband’s savings are now spent. One son is working with the same contractor that employed my husband, and I am working as a domestic help. When my husband was alive, I was not worried. There is no comparison to my situation now. A husband is a husband. I could share everything with him. I could tell him whatever happened in the family and outside. Now I have to depend on my husband’s younger brother or son to go out for some work or to meet relatives in another city.’

W6. [Urban, upper caste, 35 years old with 12 years of schooling, works as a tailor, was married for 9 years to a commission salesman, has small children]. ‘My whole world has changed. I am now living with my mother-in-law. If I have to go out I have to go with her. Whatever the circumstances, you have to learn to live with them.’

W7. [Urban, upper caste, 67 years old, holds a teaching degree, retired as a government school teacher, was married for 46 years to a government servant]. ‘There is a huge difference now. All my life I had a government job. With my husband my life was happy. I was my own mistress. Even now I am not worried economically since I have my pension, but if I need to go out I have to depend on my son. He has his own family, so he will take me only at his convenience. Who wants to depend on someone else? But now I have to live according to my son and daughter-in-law’s wishes.’

W8. [Urban, upper caste, 65 years old housewife with a master’s degree, was married to a shopkeeper for 43 years]. ‘I can hardly go out. I live with my son. He has his family and children. I hesitate to ask him… When they want to take me out they will. It is up to them.’

W9 [Urban, upper caste, 52 year old housewife with 10 years of schooling, was married to a shopkeeper for 32 years]. ‘Earlier I depended on my husband. Now I depend on my sons. They have their own families. It cannot be like it was earlier, can it? My younger son is not yet married, so if I need to go anywhere, he is the one who takes me.’

W10. [Urban, upper caste, 34 years old, has an MBA but was a housewife, was married for 5 years to a man who had a well-paid private job]. ‘Everything was fine earlier, but since the death of my husband even my own home is no longer my own. Soon after my husband’s death my mother-in-law began to fight with me. She wanted a share of the life insurance money. She was also afraid that I may ask for my share of the house, so she drove me out… I came to live with my parents. My children are small. There is corona outside. I will try and find a job once it is possible to do so.’

*Source: Contacts located and interviews conducted by Kavita Chakravarty. Survey conceptualised and excerpts translated from Hindi to English by Bina Agarwal*.

## The potential of group enterprises

It is notable that the positive COVID stories of economic survival relate to women working in groups, especially in farming. Agriculture did not face the severe national lockdown imposed on other activities in 2020, and performed better than other sectors, with a growth rate of 3.4% over the 2020–2021 financial year, even while overall GDP growth was negative at −7.3% (GoI, 2021). However this did not mean that all farmers did well. As noted earlier, large numbers of smallholders (male and female) fared poorly, with many suffering significant losses during the country-wide lockdown in 2020 due to shortages of labour for harvesting, a lack of market outlets for sales, and disruption of supply chains from farmer to consumer (Ceballos et al., 2020; Harris et al., 2020; Kulkarni et al., 2021; New Indian Express, 2020). What is striking, however, is that farmers working *in groups* survived very much better in economic terms than those cultivating individually.

The best example of this is all-women group farmers in Kerala in south India. In the early 2000s, Kerala promoted group farming as part of its anti-poverty programme, Kudumbashree (Agarwal, 2020a). Under this initiative, groups of 4–10 women lease in land and pool their labour, capital and skills to farm collectively. Today the state has 68,000 group farms, involving some 300,000 rural women. In a detailed empirical study, I compared the productivity and profitability of a sample of all-women group farms and individual family farms (95% of which were male managed) and found the women’s group farms to be significantly more productive and profitable (Agarwal, 2018, 2020b). Moreover, in early 2020, of the approximately 31,000 of these group farms that were cultivating, 87% were able to successfully harvest and sell their crops, including perishables such as fruits and vegetables (Kudumbashree, 2020a). As a group they had adequate labour for harvesting their small farms, and many sold their crops to community kitchens which were also being run by Kudumbashree women during the pandemic, and delivering some 250,000 meal packets daily to the needy in April 2020 (Outlook, 2020).

Although Kerala has some unique features in terms the state government’s commitment to a women-centric approach to development, and favourable social indicators on female literacy, sex ratios, and so on, group farming is not confined to Kerala. It has also emerged (although on a small scale) in Telangana in south India, Gujarat in the west, and Bihar and Bengal in eastern India (Agarwal, 2018, 2020b; Sugden et al., 2021). In both Gujarat (which has all-women group farms) and Bihar (which has a mixture of all-women, mixed-gender, and all-male groups) the group farmers report being more food secure than individual family farmers.^9^

Other types of groups in India have also found pathways to economic recovery under COVID-19. For instance, an estimated 66,000 members of women’s Self-Help Groups (SHGs) produced 13.2 million masks in March 2020 alone (GoI, 2020b). Many also produced hand sanitisers and protective gear (World Bank, 2020). India has 6 million women’s SHGs with 67 million members: these hold considerable potential for enabling women to earn a living by diversifying production and service activities. Working in groups also enables women to deliver on contracts and increase their bargaining power in markets. In addition, they provide women social support if needed (Agarwal, 2020b), which can be of particular help during a pandemic.

## Concluding reflections

Drawing on our understanding of pre-existing gender inequalities and social norms, and of people’s coping strategies during economic and social crises, this paper has spelt out the wide range of gender unequal effects that COVID-19 can be expected to have, and those actually measured by the telephonic surveys undertaken in India. Some of these effects could be direct, others indirect or hidden; some immediate, others long-term. We found, however, wide gaps between the effects expected and those measured in the majority of surveys. An important factor underlying these gaps was inadequate conceptualisation and learning from past crises, as well as a failure to draw effectively upon existing gender analysis. The exceptions to this are few and far between, and some are only now beginning to emerge, almost a year or more since the onslaught of the pandemic.

An analytical awareness of the potential gender effects is important when designing surveys on the pandemic, in order to track dimensions which would otherwise remain invisible, and to provide guidance for policy design for mitigating adverse effects. Media reports too provide alerts on emerging effects which surveys can follow up on. In addition, if effectively done, surveys can provide course correction to policy during a pandemic.

The Indian surveys did provide good policy leads on some counts, especially on particular relief measures such as the government’s PMJDY Rs. 500 cash transfer scheme. Several surveys found that the funds did not reach most poor women since they lacked the right bank accounts. This pointed to the need to change policy by allowing transfers into *any* bank account held by the women rather than into a specific bank account. On many other counts, however, the opportunity to provide pointers on how policy could be corrected was missed by the surveys, such as tracking the problems in the gas cylinder scheme based on reimbursements, or gender bias in the provisioning of PPE kits to women health care workers mentioned in media alerts, or the need to provide nutritional supplements rather than only grains to pregnant and lactating mothers, and so on.

Beyond the immediate, we noted the importance of tracking the loss of assets (which virtually no survey undertook), since this impinges on women’s economic situation not only directly but also indirectly, by changing intra-household power balances and increasing women’s vulnerability within families. Tracking COVID-driven poverty by gender and school dropout among girls is similarly imperative, since long-term vulnerabilities can set in which will not allow easy recovery.

In addition, existing approaches to addressing some of the much-discussed fall-outs, such as on domestic violence, need more critical scrutiny. On the reporting of domestic violence during a pandemic, for instance, the fact that many women lack access to mobile phones and internet is a serious constraint. Increasing women’s access to mobile internet could make a vast difference in reaching and supporting those at risk (apart from many other benefits that internet could bring for women). Additionally, for reducing domestic violence, enhancing women’s access to land and homesteads would open a more effective pathway than simply providing shelter homes and legal remedies, given the significantly lower risk of spousal violence faced by women owning a house or land. In fact, in 2005, India passed two landmark laws in women’s favour, one to protect women from domestic violence and the other to provide them equality in property, including the legal right of the married woman to return to her parental home and reside there (Agarwal, 2005). But the latter law is seldom evoked by women’s groups seeking to help victims of domestic violence. Here better coordination between civil society groups working on domestic violence and those working on women’s rights in land and property could make an important difference. Systematic state transfers of land to poor women, as is being done in some states, can also help.

On this and other issues, innovative group-based approaches need particular consideration. For example, government subsidies for poor women to buy land or a house in groups and share the resource could be a way forward. An example of this is the loan-cum-grant scheme launched by the Andhra Pradesh government in the 1980s for scheduled caste women to buy land in small groups and cultivate it together (Agarwal, 2003). A group approach could also work in other contexts. For instance, creating computer labs with multiple computers that have internet access in a village panchayat building, or another community building, could greatly help children who lack individual computers, especially girls, to attend online classes.

Similarly, a notable body of research across diverse social sciences shows that women working in groups are more protected and empowered economically and socially within families, markets and communities, than women on their own. And the examples I have given of women farming in groups, or SHGs producing PPE kits, indicate that the advantage of a group approach also holds during pandemics. Indeed, group approaches and community cooperation could provide new pathways both for economically recovering from this pandemic and for preparing better for a future crises.

## Footnotes

1 For example, in the recently published special issue on COVID of the journal *Feminist Economics* (2021), women’s unpaid care work was the single most important focus of 60% of the 20 articles. In another journal *Feminist Studies* (2020), which also devoted a special issue to the impact of the pandemic, women’s care work was again the single most important topic, discussed in 56% of the 16 articles.

2 Also some reports suggest there could be undercounting of Covid-related female deaths in India (Mint, 2020).

3 Excess mortality relates to deaths over and above those observed in a normal (pre-pandemic) year.

4 See also Acosta and Evans (2020), which provides a good example of prior conceptualisation in relation to girls’ education and what to expect.

5 The surveys listed in this table are not identical to the 23 surveys listed in Table A1 of Agarwal (2021b). The focus in Table 1 of the current paper is on issues covered.

6 Smaller studies point in the same direction, namely that a much smaller proportion of women than men who were earlier in paid work had recovered as of October 2020 (Dalberg, 2021).

7 SEWA is a trade union of poor, self-employed women workers that was founded in Gujarat (west India) in 1972 and has since expanded across India. SEWA-Bharat constituted in 1984 is a national federation of SEWA organizations.

8 The issue of cropping pattern has also been noted in other (ungendered) studies on India. One study on two states found that poor farmers in Odisha state who grew a diversity of crops were more food secure during the lockdown than economically better-off farmers in Haryana whose cropping patterns were more monocultural (Ceballos et al., 2020).

9 Personal communication in May 2020 by those working with these groups, viz. Hiral Dave, Cohesion, for Gujarat, and Anoj Kumar, International Water Management Institute, for Bihar.
